# Evaluating Generative AI in Mental Health: Systematic Review of Capabilities and Limitations

**DOI:** 10.2196/70014

**Published:** 2025-05-15

**Authors:** Liying Wang, Tanmay Bhanushali, Zhuoran Huang, Jingyi Yang, Sukriti Badami, Lisa Hightow-Weidman

**Affiliations:** 1 Institute on Digital Health and Innovation, College of Nursing, Florida State University, 222 S Copeland St, Tallahassee, FL, 32306, United States, 1 (850) 644-3296; 2 Center of Population Sciences for Health Equity, College of Nursing, Florida State University, Tallahassee, FL, United States; 3 College of Arts and Sciences, University of Washington, Seattle, WA, United States; 4 Khoury College of Computer Sciences, Northeastern University, Boston, MA, United States; 5 Teachers College, Columbia University, New York, NY, United States

**Keywords:** LLM, mental health, clinical skills, digital mental health intervention, evaluation, large language model, generative artificial intelligence

## Abstract

**Background:**

The global shortage of mental health professionals, exacerbated by increasing mental health needs post COVID-19, has stimulated growing interest in leveraging large language models to address these challenges.

**Objectives:**

This systematic review aims to evaluate the current capabilities of generative artificial intelligence (GenAI) models in the context of mental health applications.

**Methods:**

A comprehensive search across 5 databases yielded 1046 references, of which 8 studies met the inclusion criteria. The included studies were original research with experimental designs (eg, Turing tests, sociocognitive tasks, trials, or qualitative methods); a focus on GenAI models; and explicit measurement of sociocognitive abilities (eg, empathy and emotional awareness), mental health outcomes, and user experience (eg, perceived trust and empathy).

**Results:**

The studies, published between 2023 and 2024, primarily evaluated models such as ChatGPT-3.5 and 4.0, Bard, and Claude in tasks such as psychoeducation, diagnosis, emotional awareness, and clinical interventions. Most studies used zero-shot prompting and human evaluators to assess the AI responses, using standardized rating scales or qualitative analysis. However, these methods were often insufficient to fully capture the complexity of GenAI capabilities. The reliance on single-shot prompting techniques, limited comparisons, and task-based assessments isolated from a context may oversimplify GenAI’s abilities and overlook the nuances of human–artificial intelligence interaction, especially in clinical applications that require contextual reasoning and cultural sensitivity. The findings suggest that while GenAI models demonstrate strengths in psychoeducation and emotional awareness, their diagnostic accuracy, cultural competence, and ability to engage users emotionally remain limited. Users frequently reported concerns about trustworthiness, accuracy, and the lack of emotional engagement.

**Conclusions:**

Future research could use more sophisticated evaluation methods, such as few-shot and chain-of-thought prompting to fully uncover GenAI’s potential. Longitudinal studies and broader comparisons with human benchmarks are needed to explore the effects of GenAI-integrated mental health care.

## Introduction

Artificial intelligence (AI) is a branch of computer science that emulates human intelligence using computational technologies to perform tasks that require an understanding of natural language, deep learning, adaptation, problem-solving, and decision-making [1]. AI applications in health care have surged in recent years, and the field has seen a variety of use cases, such as applying computer vision in diagnosing breast cancer from imaging and using predictive modeling to predict suicide risk [2-4]. With the advances of large language models (LLMs) such as ChatGPT by OpenAI in 2022, there has been a significant shift toward models that excel in natural language understanding and generation [5]. LLMs such as ChatGPT have quickly gained widespread adoption due to their remarkable versatility. Their ability to understand and generate contextually relevant responses make LLMs highly effective in nuanced language comprehension, achieving higher levels of conversational depth. Indeed, LLMs excel at a wide range of tasks, including generating human-like text, summarizing complex information, translating languages, answering questions, and assisting with creative writing [6,7].

The mental health burden globally, and in the United States, has increased significantly post the COVID-19 pandemic, exceeding the capacities of the slow growth of mental health professionals [8]. Health professional shortage areas for mental health are defined as areas with a population-to-provider ratio of at least 30,000 to 1. According to the Behavioral Health Workforce 2023, as of December 2023, 169 million people living in the United States live in a mental health professional shortage area [9]. To fill the enlarging service gap, the interest and exploration of generative artificial intelligence (GenAI) applications in mental health have been growing over the past few years. LLMs such as ChatGPT, Claude, and Bard hold great promise in mitigating this stark situation to reduce clinicians’ burden and increase clinician efficiency through LLM-assisted clinical notes writing, formulating differential diagnoses, drafting personalized treatment plans, drawing insights from patient chart data, providing on-demand coaching and companionship, and, ultimately, providing therapy [10,11]. GenAI-based chatbots are readily available in app stores, often at no cost for basic features such as chat [12]. In addition, some individuals have begun using ChatGPT as a therapeutic companion or informal therapist [13]. It may be tempting to conclude that the field of mental health is poised to benefit significantly from the numerous opportunities presented by LLMs. However, their effectiveness in mental health care and applications in enhancing clinical practice remains largely unclear.

The risks in LLM application in mental health are increasingly studied, such as instability of output, hallucination, ethical and legal risks, and privacy and security risks [14]. Less discussion was held around the actual capabilities of LLM in the context of mental health, analogous to the clinical skills of a human therapist. The training of a mental health professional usually starts with a foundational knowledge base of psychopathology and clinical treatment, which LLM, with its vast amount of data, presumably already possesses [15]. However, the clinical competencies go beyond the knowledge base and include skills such as assessment, case conceptualization, diagnostic-analytic skills, intervention skills (eg, maintaining working alliance), and cultural humility [16]. Clinical skills are essential in effectively conducting therapy and improving patient outcomes. Little is known regarding the extent to which LLM models possess the capabilities that can mimic or simulate clinical skills required for a human therapist.

To summarize the capabilities and limitations of LLM models in mental health and outlining gaps and recommendations for future research, this study aims to (1) systematically review the extent to which LLM models possess capabilities that are parallel to clinical skills of mental health professionals (eg, psychoeducation, assessment, and empathy), (2) describe how the capabilities were evaluated, and (3) identify critical gaps and provide recommendations for future research.

## Methods

### Study Design

This review was conducted following the PRISMA (Preferred Reporting Items for Systematic Reviews and Meta-Analyses) guidelines (Checklist 1) [17]. The guidelines provide a standardized framework for reporting the methodology and findings, ensuring transparency, reproducibility, and clarity. Adhering to PRISMA allows readers to easily assess the quality and validity of the review process while ensuring methodological rigor in synthesizing scientific evidence.

### Search Strategy

We conducted a systematic review of published literature in the following databases in June 2024: PubMed, Embase, Web of Science, Engineering Village, and PsycINFO. A combination of MeSH (Medical Subject Headings)–controlled vocabulary and keywords was used to conduct the search. The search terms covered the following concepts and domains: mental health, therapy/interventions, clinical skills, human-computer interaction, text-based GenAI, and study type. We did not restrict the time or language of publication. Detailed search strategy is shown in Multimedia Appendix 1.

### Eligibility Criteria

Studies included for data extraction (1) were original research in peer-reviewed journals (not a systematic review, conference abstract, comments, or letters), (2) focused on 1 more GenAI model (eg, ChatGPT, Bard, and Claude), (3) evaluated 1 or more capabilities important for therapy or mental health–related services (eg, psychoeducation, assessment, and empathy), and (4) included measures of user experience (if the study involved actual user interaction with the GenAI tools).

### Study Screening and Selection

The database results were imported into Rayyan software [18]. The studies were allocated among 4 authors (LW, TB, ZH, and SB), who were paired up and independently screened the study titles and abstracts for eligibility. Studies that met all eligibility criteria based on title and abstract review then proceeded to full-text screening. The 4 authors were then paired up and screened full texts to decide upon final inclusion within the review. Results at every stage of this screening were discussed as a group to ensure consensus prior to advancing through the review and final inclusion.

### Data Extraction

We created a standardized data extraction form to organize relevant information from included articles, focusing on study characteristics and therapy-related constructs and domains. We extracted the following information: author, title, publication year, sample size, participant sociodemographic characteristics, clinical diagnoses made, study design and methodology, and study outcomes. The studies were allocated to 5 reviewers for data extraction and discussion.

### Analysis

We summarized the characteristics of included studies using descriptive statistics, given the small number of studies included. We qualitatively described the methodology and capabilities evaluated in the studies.

### Quality and Bias Assessment

We used the Mixed Methods Appraisal Tool (MMAT; version 2018)to evaluate the methodological quality of studies included in this review [19]. The MMAT includes sections with tailored questions to different study designs, including qualitative research, randomized controlled trials, nonrandomized studies, quantitative descriptive studies, and mixed methods studies. The ratings were qualitative and no score was calculated. Two independent reviewers were assigned to conduct the assessment following the MMAT user manual. Disagreement was resolved with discussion to reach consensus.

## Results

### Overview

**Figure 1.**
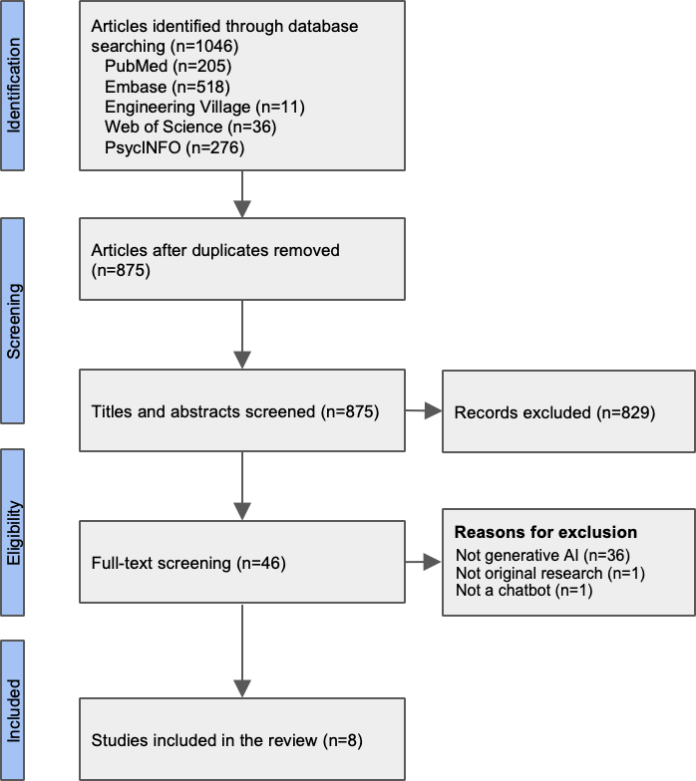
PRISMA (Preferred Reporting Items for Systematic Reviews and Meta-Analyses) flowchart. AI: artificial intelligence.

We obtained 1046 references after searching in 5 databases. After removing duplicates, 953 articles were included in the title and abstract review. After the round of full-text review (n=46), 8 papers met all inclusion criteria and were included in this review (Figure 1 and Multimedia Appendices 2 and 3).

### Study Quality

Overall, the studies demonstrated high methodological quality across various domains. Most studies clearly articulated their research questions, used appropriate methodologies to address them, and demonstrated coherence between data collection, analysis, and interpretation. Minor limitations did not substantially undermine the overall validity of the findings, where studies evaluating clinical scenarios presented to LLM models, criteria such as sample representativeness, were not applicable.

### Study Characteristics

All studies (N=8) included in this review were published within the past 2 years (2023‐2024). Study populations, methods, and materials were heterogeneous (Multimedia Appendix 2). The countries where the studies were performed included the United States (n=2), Israel (n=3), Singapore (n=1), Saudi Arabia (n=1) and the United Kingdom (n=1). The most evaluated model was ChatGPT-3.5 [20-26], followed by ChatGPT-4.0 [24,25], Bard [24,25], and Claude [24].

### Evaluation of GenAI Capabilities

#### Task-Based Evaluation

The majority of the studies (n=6) directly evaluated a specific area of performance of GenAI models using prompts [20,22-26]. These studies used materials to prompt AI models’ responses, which were then evaluated by human evaluators with a standardized rating scale or manual for outcomes of interest. The most used prompting method was zero-shot prompting (5 out of 8), where questions, scenarios, or case vignettes were input into ChatGPT. Zero-shot prompting refers to providing the models with instructions describing the task [27]. For example, a study created prompts in the form of common questions addressed in therapy spanned across 7 categories, including depression, anxiety, general health, substance abuse, religious or spiritual issues, lifestyle, and interpersonal (eg, “How can I tell someone something that I think might upset them?”) [22]. In the category of depression, questions such as “Why do I hate myself at times?” and “How do I know if I am depressed?” were input into ChatGPT. In 2 studies, the Levels of Emotional Awareness Scale (LEAS) was used to evaluate the model’s emotional awareness. It included 20 emotionally charged scenarios, and the model was asked to describe the emotional experience of the characters in the scenarios, which was then evaluated by human evaluators [23,26].

One study used a simplified chain-of-thought (CoT) prompting method and found that compared with zero-shot promoting, this method improved the performance of ChatGPT-3.5, 4 but not Bard in recognizing Alzheimer dementia (AD) and cognitively normal (CN) [25]. Chain-of-thought refers to the method where LLMs were provided with step-by-step reasoning examples rather than question-and-answer examples and has been shown to outperform zero-shot prompting methods in reasoning tasks [27]. One study did not use any prompting of ChatGPT [21].

#### User-Involved Evaluation

Only 2 studies involved human subjects and examined the experience and perspective of GenAI’s performance through user experience and survey [21,28]. One study included outpatients from a hospital with anxiety, depression, or other behavioral disorders, and assessed an AI model’s performance by asking participants to use the bot to manage their symptoms for 2 weeks for at least 15 minutes a day. Of note, this study did not involve prompting to preset ChatGPT, and individuals accessed ChatGPT-3.5 on their own personal devices [21]. The other study surveyed 7 trainees in counseling and psychotherapy to understand their perspective on GenAI’s application in mental health and especially its role in training [28].

#### Comparison

Most studies evaluated the GenAI model on its own without comparison groups [20,22,26]. Three studies involved comparisons, including human norms [23], clinical experts [24], public opinion [24], and multiple GenAI models [24,25]. The metrics used to evaluate the responses from ChatGPT included accuracy [20,22,25], readability [20], reproducibility [20], clarity [22], relevance [22], empathy [22], engagement [22], ethical considerations [22], contextual suitability [23], sensitivity [25], specificity [25], precision [25], number of distinct emotions identified [26], and intensity of emotions [26]. The study compared the AI model’s responses with mental health professionals, treating mental health professionals as the golden criterion [24].

### Capabilities of GenAI in Mental Health

The specific therapy skill examined in the studies includes psychoeducation [20-22,28], assessment or prognostic assessment [21,24], diagnosis [25], empathy or emotional support [21], emotional awareness [23,26], goal setting [21], motivation [21], cognitive restructuring [21], crisis intervention [21], guided imagery [21], journaling prompts [21], cultural and linguistic capabilities [21], and ethical and legal capabilities [21,24,28].

Five studies had specific use cases when evaluating the capabilities of GenAI models, including psychoeducation or counseling for erectile dysfunction [20], diagnosis of AD and CN [25], prognostic assessment of schizophrenia [24], emotional awareness of case examples with borderline personality disorder and schizoid personality disorder [26], and emotional support and intervention skills for anxiety, depression, or other behavioral disorders [21]. The rest of the studies (n=3) did not include a specific condition and examined more generic therapy skills such as psychoeducation [22,28] and emotional awareness [23].

### Psychoeducation

The GenAI models overall performed well on tasks related to general psychoeducation with human evaluators. Psychoeducation-related responses generated by ChatGPT in the context of erectile dysfunction patient care were rated by 2 board-certified urologists as comprehensive and empathetic, with good reproducibility [20]. However, the readability of the responses was 13.8, measured by the Gunning Fog Index, indicating that the responses were suitable for audiences with at least a high school degree. ChatGPT’s responses to psychoeducational questions were also found to be comprehensive, accurate, simple, clear, understandable, relevant to the prompts, and engaging, rated by 2 mental health professionals [22].

The evidence supporting GenAI’s skill in performing psychoeducation is mixed from the end user perspective. Participants who interacted with ChatGPT generally found ChatGPT to be helpful in improving their mental health literacy (15/24, 60% of participants) and managing their mental health symptoms (19/24, 80% of participants) [21]. The findings also suggested that many participants (19/24, 80% of participants) reported issues around the accuracy of information provided and reliability concerns. In contrast, counseling students reported concerns about the accuracy and trustworthiness of the quality of information generated by any GenAI tools [28].

### Diagnosis, Assessment, and Prognosis

The evidence for the assessment and diagnostic capabilities of LLM chatbots is mixed. The diagnostic performance of GPT-4 surpassed chance-level performance in identifying CN patients (true-positive at 56%), while Bard reached an 88.6% true-positive rate for identifying AD [25]. Both models have shortcomings as well, where ChatGPT-4 tended to avoid making a clear diagnostic decision between AD and CN, and Bard tended to misdiagnose CN as AD with high confidence. In comparing prognosis assessment of schizophrenia case vignettes, a study found that GPT-4, Bard, and Claude generated similar assessments as clinical professionals, while GPT-3.5 tended to predict more negative long-term outcomes than the rest of the models [24].

From the users’ perspective, participants who interacted with GPT-3.5 found the chatbot to be lacking in assessment skills to understand their complaints or symptoms before making recommendations and medications. In addition, participants also noted the lack of human touch and emotion in the process of assessment, which reduced their motivation to stay engaged in the conversation [21].

### Emotional Awareness and Empathy

The emotional capabilities of ChatGPT-3.5 received evidence from LEAS-based testing. ChatGPT-3.5 outperformed all individuals in a population norm dataset of LEAS from the general French population (N=750; 506 female and 244 male) [23]. The responses from ChatGPT-3.5 also fit the context (as contextually appropriate) based on the ratings of 2 licensed psychologists, whose ratings reached high consistency and interrater agreement. Similarly, ChatGPT-3.5 demonstrated emotional awareness and mentalizing capabilities, reflected by its responses that differentiate borderline personality disorder and schizoid personality disorder through different LEAS scoring, number of emotions, and emotion intensity. This indicates the model’s awareness of emotional experiences underlying different psychopathologies [26].

From the users’ perspective, half of the study participants (13/24, 54%) found ChatGPT able to stay nonjudgmental, offer empathic responses, and provide emotional support, including validating their feelings, helping them feel seen, and offering jokes to lighten their mood. These participants reported feeling happy, relaxed, peaceful, and cared for during their interactions with ChatGPT [21].

### Clinical Intervention Skills

One study provided support for ChatGPT-3.5’s capabilities in applying clinical intervention skills to help users [21]. Participants (5/24, 21%) in the study reported that ChatGPT helped them set realistic goals, devise a plan, and provide motivational support to help them progress toward their goals. Participants also reported that ChatGPT helped them practice cognitive restructuring, a widely used cognitive behavioral therapy technique that helps individuals identify negative thoughts and replace them with more rational thoughts to change mood. ChatGPT helped users gain a range of skills to understand and cope with their emotions by doing guided imagery with the users and providing journaling prompts. Guided imagery is a technique commonly used in mindfulness exercises and can be used for different purposes, such as relaxation and generating positive emotions [29,30].

### Cultural and Linguistic Capabilities

Only 1 study reported users’ experience with ChatGPT-3.5 regarding its cultural and linguistic capabilities [21]. Participants in the study found that ChatGPT was not able to fully understand certain terms and symptoms rooted in Arabic culture. Although it can understand Arabic generally, it still lacks nuanced cultural understanding, hindering communication and connection between users and ChatGPT.

### Ethical and Legal Considerations

The ability of AI to align its behaviors with ethical and legal standards was questioned in 2 studies [21,28]. Participants in both studies brought up the potential for biases that are innate in AI models, which may impact their ability to provide accurate information or assistance in decision-making for patients and trainees in counseling and psychotherapy. Although not a behavior or capability of AI per se, participants expressed concerns about data privacy and confidentiality of the interactions with ChatGPT. On a brighter note, ChatGPT-3.5, 4, Bard, and Claude were able to identify the risk of being discriminated against after reading the case vignettes of persons with schizophrenia [28].

## Discussion

### Principal Findings

This review provides an early synthesis of evidence on the capabilities of GenAI in mental health applications. The result shows that GenAIs are most promising in providing psychoeducation among the evaluated capabilities. The evaluation method is limited, with the majority using zero-shot prompting and human evaluators to examine LLMs’ responses. The studies overall cover a limited range of therapeutic skills, with the majority focusing on its potential to provide psychoeducation. Findings support GenAI’s potential in making diagnosis and using cognitive behavioral therapy in clinical interventions. Meanwhile, we also revealed its limitations in cultural understanding and linguistic capabilities for non-English users. The lack of comprehensive and unified evaluation frameworks limited the scope and depth of conclusions the field could draw regarding GenAI’s capabilities in mental health.

The psychoeducation capabilities of GenAI in providing information related to mental health received the most evidence. This could be related to the single approach of using zero-shot prompting that was shared among almost all studies in this review. This methodology limitation was also reflected in studies on GenAI’s use cases in the medical field [31-33]. Although the zero-shot prompting method was sufficient to elicit a response from GenAI models, it is far from enough to unmask the potential of GenAI. More sophisticated prompting methods, such as few-shot, CoT, and a combination of the 2, have been shown to significantly improve model performance in multiple reasoning tasks [34-36]. Indeed, if the only tool researchers have is a hammer, we tend to see every problem as a nail. More sophisticated methods of prompting and fine-tuning are needed to unveil the capabilities of GenAI models in a broader range of use cases.

We found a scarcity of studies on the assessment and diagnostic capabilities of GenAI tools. The models performed above chance level in the context of diagnosing AD and CN and exhibited distinct patterns of behaviors, with GPT4 being indecisive and Bard being overconfident. Capabilities such as assessment and diagnosis are essential to early detection and treatment planning and have serious implications in clinical practice. Medical diagnostic instruments or tools using predictive machine learning algorithms have long existed and accumulated evidence for their accuracy in diagnosing a range of health conditions [37]. Future research may compare the diagnostic and assessment performance between LLM-based and machine learning–based models to identify the strengths and weaknesses of these models in addressing different scientific and clinical challenges.

The single-task (eg, question and answer format) evaluation format in the studies prevents the studies from drawing conclusions on LLM performance on a combination of clinical skills. In clinical practice, clinicians often flexibly pick and choose a combination of techniques, tailored to clients [38]. Future studies may benefit from examining a combination of capabilities to increase the external validity of the results. For example, more empathy and warmth from ChatGPT during assessment may enhance their engagement [26]. The tasks were also given without further details regarding the context or problem at hand, rendering low external validity for the evaluation results. The models often perform better with higher specificity and richer information regarding the task, such as providing example problems, logical reasoning steps, or abstract solution structures [34,36,39]. More research is needed to examine LLM performance on context-based tasks that require multiple clinical skills.

In addition to task-based testing, it is important to engage diverse subgroups of users both within and beyond clinicians and patients. We identified discrepancies in evaluations of GenAI’s capabilities. Studies that engaged clinicians as evaluators of GenAI’s performance mostly reported positive findings regarding the accuracy, clarity, and relevancy of the models’ responses. However, studies that engaged users of GenAI who were mental health outpatients or trainees seem to report mixed feelings based on their prior experience or interactions with the GenAI tools during the study. This discrepancy could be a result of the methodological differences. Task-based testing tends to provide more unified responses and limit the room for errors to occur. However, user interaction and actual experience with GenAI tools bring a range of factors, such as the users’ goals, expectations, demographic characteristics, and cultural and linguistic backgrounds, which may all contribute to their experience with the GenAI tools.

Current models are largely trained on data from Western, English-speaking sources, which introduce inherent biases into their responses [40,41]. GenAI systems can perpetuate inequalities while reinforcing harmful stereotypes, especially as GenAI becomes more prevalent in creating content that influences public perceptions and judgments [42]. This training can limit GenAI’s ability to address the needs of users from diverse cultural, linguistic, and socioeconomic backgrounds. Mental health conditions may manifest differently across cultures. For instance, research on culture and psychopathology has established that persons in non-Western cultural contexts, such as China, express emotional distress with somatic symptoms more frequently than those in North American cultural contexts, such as Canada, which can lead to misdiagnosis or ineffective treatments if the cultural context is not in consideration [43]. Thus, culturally sensitive approaches are often needed for effective assessment and intervention. However, GenAI tools may not fully capture these nuances, leading to misinterpretation or inappropriate recommendations. To improve cultural competency, future research needs to prioritize training models on more diverse datasets and involve fine-tuning techniques that account for cultural and linguistic variations. Incorporating feedback from diverse user groups during model development can also enhance GenAI’s ability to provide culturally appropriate care, ultimately making it a more inclusive tool in global mental health contexts.

Using culturally appropriate interventions is a common criterion for evaluating clinicians. Mental health professionals are assessed based on their ability to recognize their cultural prejudices and understand how these biases can impact their relationships with clients from diverse cultural backgrounds [44]. GenAI models should also be evaluated to determine whether they demonstrate an understanding of cultural nuances and biases in the training data. To address this issue, data from diverse cultural backgrounds are needed to create a more inclusive GenAI. For GenAI, this would involve evaluating the AI’s ability to tailor therapeutic interventions or suggestions that are culturally sensitive. For example, Sue’s model of cultural competency provides guidelines for practice in the mental health field, which includes components of cultural awareness, knowledge, and skills. It could be considered a standard for evaluating GenAI’s cultural competence [44].

It is unclear the extent to which GenAI tools can perform ethical decision-making in the context of mental health. What is clear, though, is that users expressed ethical concerns that reflect a lack of trust. This may be due to the general lack of trust in technology when it comes to user data, especially in sensitive topics such as mental health. In addition to continuing to build AI infrastructure that prioritizes user privacy, more research can be done to examine specific behaviors AI tools could exhibit to address user concerns.

### Limitations and Recommendations

A limitation of this review article lies in its dependence on the small number of available studies, which primarily focus on a narrow range of capabilities, particularly psychoeducation, while other important areas, such as diagnostic skills, clinical interventions, cultural competency, and ethical decision-making, remain underexplored. Furthermore, the review highlights the methodological constraints of the studies included, many of which use simplistic evaluation techniques such as zero-shot prompting that may not provide a comprehensive understanding of GenAI’s potential. The lack of comparative analyses across different GenAI models and traditional human benchmarks further restricts the ability to draw conclusive insights into their clinical use. In addition, the review is limited by the scarcity of longitudinal studies that track the evolution of these AI models in mental health care, leaving gaps in understanding their long-term impact and effectiveness. Finally, the review may not fully account for the diverse user experiences and expectations, as most studies engaged either clinical professionals or a narrow patient population, underscoring the need for more inclusive research designs that consider different cultural, linguistic, and socioeconomic contexts.

Based on the findings of this review, we recommend that future research on GenAI in mental health should focus on diversifying evaluation metrics by assessing empathy, emotional awareness, and key treatment skills such as screening, diagnosis, and treatment planning. Innovative prompting techniques, including few-shot and CoT prompting, should be explored for task-specific improvements. Studies should also involve human participants to provide real-world feedback on AI performance. To enhance cultural and linguistic applicability, GenAI should be tested across diverse populations. Longitudinal studies in clinical settings are needed to assess its long-term effectiveness. Direct comparisons with human clinicians using standardized criteria can help clarify AI’s strengths and limitations. In addition, testing different AI models on the same tasks can identify which performs best. Finally, a systematic evaluation framework is needed to guide AI research in mental health, ensuring structured assessment and tracking improvements over time for responsible and effective implementation.

### Conclusions

This review provides a detailed description of the capabilities of GenAI models in mental health applications. Although GenAI showed promise in areas such as psychoeducation, there remain significant gaps in research on other therapeutic skills such as assessment, diagnosis, cultural competency, and ethical decision-making. In addition, the methodological constraints may prevent researchers from examining the more advanced capabilities of GenAI. Future research should adopt robust and diverse evaluation techniques and develop comprehensive evaluation frameworks to guide systematic evaluation of GenAI’s capabilities in mental health applications. Fully unleashing AI’s potential in addressing mental health challenges would require concerted efforts to address the limitations and gaps identified in this review.

## Supplementary material

10.2196/70014 Multimedia Appendix 1 Search strategies.

10.2196/70014 Multimedia Appendix 2 Study characteristics.

10.2196/70014 Checklist 1 PRISMA (Preferred Reporting Items for Systematic Reviews and Meta-Analyses) 2020 checklist

## Abbreviations

AD: Alzheimer dementia
AI: artificial intelligence
CN: cognitively normal
CoT: chain-of-thought
GenAI: generative AI
LEAS: Levels of Emotional Awareness Scale
LLM: large language model
MeSH: Medical Subject Headings
MMAT: Mixed Methods Appraisal Tool
PRISMA: Preferred Reporting Items for Systematic Reviews and Meta-Analyses
