# Supplementary material for: Evaluating Generative AI in Mental Health: Systematic Review of Capabilities and Limitations
Source: JMIR Ment Health. 2025 May 15;12:e70014. doi: 10.2196/70014 (PMC12097452; doi:10.2196/70014)
Supplement: Multimedia Appendix 1 [file mental-v12-e70014-s001.docx]

**Multimedia Appendix 1: Search Strategies**

| **Concepts** | **Keywords** | **Controlled Vocabulary** | **Search string** |
| --- | --- | --- | --- |
| Mental health | "mental health"[Title/Abstract] OR depression[Title/Abstract] OR "psychological distress"[Title/Abstract] OR "anxiety"[Title/Abstract] OR "PTSD"[Title/Abstract] OR  “Bipolar”[Title/Abstract] OR  “Disorder”[Title/Abstract] OR  "Post-traumatic stress disorder"[Title/Abstract] OR "stress"[Title/Abstract] OR suicid*[Title/Abstract] OR  sleep[Title/Abstract] OR  insomnia[Title/Abstract] OR  “cognitive impairment” [Title/Abstract] OR  “neurocognitive impairment” [Title/Abstract] OR   “neurological complication” [Title/Abstract] OR  Dementia [Title/Abstract] OR  loneliness [Title/Abstract] | "Mental Health"[Mesh] OR "Depression"[Mesh] OR  "Depressive Disorder"[Mesh] OR "Psychological Distress"[Mesh] OR "suicide"[Mesh]OR  "anxiety"[Mesh] OR  “Stress Disorders, Post-Traumatic"[Mesh] OR  “sleep”[Mesh] OR  “Sleep Initiation and Maintenance Disorders”[Mesh] OR “Dementia”[Mesh] OR  “Loneliness”[Mesh] OR  "Mood Disorders"[Mesh] OR "Substance-Related Disorders"[Mesh]OR "Psychology"[Mesh] OR  "Psychiatry"[Mesh] | "mental health"[Title/Abstract] OR depression[Title/Abstract] OR "psychological distress"[Title/Abstract] OR "anxiety"[Title/Abstract] OR "PTSD"[Title/Abstract] OR  “Bipolar”[Title/Abstract] OR  “Disorder”[Title/Abstract] OR  "Post-traumatic stress disorder"[Title/Abstract] OR "stress"[Title/Abstract] OR suicid*[Title/Abstract] OR  sleep[Title/Abstract] OR  insomnia[Title/Abstract] OR  “cognitive impairment” [Title/Abstract] OR  “neurocognitive impairment” [Title/Abstract] OR   “neurological complication” [Title/Abstract] OR  Dementia [Title/Abstract] OR  loneliness [Title/Abstract] OR  "Mental Health"[Mesh] OR "Depression"[Mesh] OR  "Depressive Disorder"[Mesh] OR "Psychological Distress"[Mesh] OR "suicide"[Mesh]OR  "anxiety"[Mesh] OR  “Stress Disorders, Post-Traumatic"[Mesh] OR  “sleep”[Mesh] OR  “Sleep Initiation and Maintenance Disorders”[Mesh] OR “Dementia”[Mesh] OR  “Loneliness”[Mesh] OR  "Mood Disorders"[Mesh] OR "Substance-Related Disorders"[Mesh]OR "Psychology"[Mesh] OR  "Psychiatry"[Mesh] |
| Therapy related/intervention | "Behavioral coach*"[Title/Abstract] OR  "psychotherap*"[Title/Abstract] OR  "Psychotherapy"[Title/Abstract] OR  "Behavioral therapy"[Title/Abstract] OR  "Behavioral assessment"[Title/Abstract] OR  "Cognitive behavioral therapy"[Title/Abstract] OR  "Dialectical Behavior Therapy"[Title/Abstract] OR  "Interpersonal Therapy"[Title/Abstract] OR  "Rational emotive behavior therapy"[Title/Abstract] OR  "Mindfulness-Based Therapies"[Title/Abstract] OR  "Exposure Therapy"[Title/Abstract] OR  "Acceptance and Commitment Therapy"[Title/Abstract] OR  "Family Therapy"[Title/Abstract] OR  "Cognitive Processing Therapy"[Title/Abstract] | "Counseling"[Mesh] OR  "Mental Health Services"[Mesh] OR  Psychotherapy[Mesh] OR  “Behavior therapy”[Mesh] OR  Neuropsychological Tests[Mesh] OR  “Dialectical Behavior Therapy” [Mesh] OR  “Family therapy”[Mesh] OR  "Acceptance and Commitment Therapy” [Mesh] | "Behavioral coach*"[Title/Abstract] OR  "psychotherap*"[Title/Abstract] OR  "Psychotherapy"[Title/Abstract] OR  "Behavioral therapy"[Title/Abstract] OR  "Behavioral assessment"[Title/Abstract] OR  "Cognitive behavioral therapy"[Title/Abstract] OR  "Dialectical Behavior Therapy"[Title/Abstract] OR  "Interpersonal Therapy"[Title/Abstract] OR  "Rational emotive behavior therapy"[Title/Abstract] OR  "Mindfulness-Based Therapies"[Title/Abstract] OR  "Exposure Therapy"[Title/Abstract] OR  "Acceptance and Commitment Therapy"[Title/Abstract] OR  "Family Therapy"[Title/Abstract] OR  "Cognitive Processing Therapy"[Title/Abstract] OR  "Counseling"[Mesh] OR  "Mental Health Services"[Mesh] OR  Psychotherapy[Mesh] OR  “Behavior therapy”[Mesh] OR  Neuropsychological Tests[Mesh] OR  “Dialectical Behavior Therapy” [Mesh] OR  “Family therapy”[Mesh] OR  "Acceptance and Commitment Therapy” [Mesh] |
| Specific clinical skills | “Therapeutic alliance”[Title/Abstract] OR  “Cultural competency” [Title/Abstract] OR  “Assessment” [Title/Abstract] OR  “Data collection” [Title/Abstract] OR  “Conceptualization” [Title/Abstract] OR  “Case formulation” [Title/Abstract] OR  “Reflect” [Title/Abstract] OR  “Summary” [Title/Abstract] OR  “Motivation” [Title/Abstract] OR  “behavioral goals”[Title/Abstract] OR  “Goal setting”[Title/Abstract] OR  “Foster hope” [Title/Abstract] OR  “Theor*” [Title/Abstract] OR  “*Ethic*” [Title/Abstract] OR  “Metaphor*” [Title/Abstract] OR  “empath*”[Title/Abstract] OR  “emotion*”Title/Abstract] | “Therapeutic alliance”[Mesh] OR  “motivation”[Mesh] OR  “Data collection” [Mesh] OR  “empathy”[Mesh] OR  Ethics [Mesh] OR  “Psychological Theory” [Mesh] OR  “Emotions” [Mesh] | “Therapeutic alliance”[Title/Abstract] OR  “Cultural competency” [Title/Abstract] OR  Assessment [Title/Abstract] OR  “Data collection” [Title/Abstract] OR  Conceptualization [Title/Abstract] OR  “Case formulation” [Title/Abstract] OR  “*Reflect” [Title/Abstract] OR  “Summary” [Title/Abstract] OR  “*Motivation” [Title/Abstract] OR  “behavioral goals”[Title/Abstract] OR  “Goal setting”[Title/Abstract] OR  “Foster hope” [Title/Abstract] OR  “Theor*” [Title/Abstract] OR  “*Ethic*” [Title/Abstract] OR  “Metaphor*” [Title/Abstract] OR  “empath*”[Title/Abstract] OR  “Therapeutic alliance”[Mesh] OR  “motivation”[Mesh] OR  “Data collection” [Mesh] OR  “empathy”[Mesh] OR  “Ethics” [Mesh] OR  “Psychological Theory” [Mesh] OR  “Emotions” [Mesh] |
| HCI | “Trust” [Title/Abstract] OR  “security” [Title/Abstract] OR  “accuracy” [Title/Abstract] OR  “Privacy” [Title/Abstract] OR  “Connect*” [Title/Abstract] OR  “Human-computer interaction” [Title/Abstract] OR  “User experience” [Title/Abstract] OR  “User interface” [Title/Abstract] OR  “Usability” [Title/Abstract] OR  “User engagement” [Title/Abstract] OR  “User adherence” [Title/Abstract] OR  “User satisfaction”[Title/Abstract] OR  “Interaction design”[Title/Abstract] OR  “Conversation* design”[Title/Abstract] OR  “Anthropomorphism”[Title/Abstract] OR  “Personalization”[Title/Abstract] OR  “Adapt*”[Title/Abstract] OR  “Tailor*”[Title/Abstract] OR  “Accessibility”[Title/Abstract] OR  “User satisfaction”[Title/Abstract] OR  “User acceptance”[Title/Abstract] | “Trust”[Mesh] OR  “Data Accuracy”[Mesh] OR  “privacy”[Mesh] OR  “User-Computer Interface”[Mesh] OR  “User-Centered Design”[Mesh] OR  “Patient satisfaction” [Mesh] | “Trust” [Title/Abstract] OR  “security” [Title/Abstract] OR  “accuracy” [Title/Abstract] OR  “Privacy” [Title/Abstract] OR  “Connect*” [Title/Abstract] OR  “Human-computer interaction” [Title/Abstract] OR  “User experience” [Title/Abstract] OR  “User interface” [Title/Abstract] OR  “Usability” [Title/Abstract] OR  “User engagement” [Title/Abstract] OR  “User adherence” [Title/Abstract] OR  “User satisfaction”[Title/Abstract] OR  “Interaction design”[Title/Abstract] OR  “Conversation* design”[Title/Abstract] OR  “Anthropomorphism”[Title/Abstract] OR  “Personalization”[Title/Abstract] OR  “Adapt*”[Title/Abstract] OR  “Tailor*”[Title/Abstract] OR  “Accessibility”[Title/Abstract] OR  “User satisfaction”[Title/Abstract] OR  “User acceptance”[Title/Abstract] OR  “Trust”[Mesh] OR  “Data Accuracy”[Mesh] OR  “privacy”[Mesh] OR  “User-Computer Interface”[Mesh] OR  “User-Centered Design”[Mesh] OR  “Patient satisfaction” [Mesh] |
| genAI (text))  -DALL-E not included as it’s picture | “chatgpt*” [Title/Abstract] OR  "chat generative" [Title/Abstract] OR  "generative AI" [Title/Abstract] OR  “artificial intelligence” [Title/Abstract] OR  “AI” [Title/Abstract] OR  "generative artificial intelligence" [Title/Abstract] OR  “GPT” [Title/Abstract] OR  "Generative Pretrained Transformer" [Title/Abstract] OR  “chatbot*” [Title/Abstract] OR   "Large language model*" [Title/Abstract] OR  "AI language model*" [Title/Abstract] OR  "artificial intelligence language model*" [Title/Abstract] OR  "conversational agent*" [Title/Abstract] OR  "Natural language generation" [Title/Abstract] OR  “NLG” OR "Text generation" [Title/Abstract] OR  "text synthesis" [Title/Abstract] OR  "Transformer language model*" [Title/Abstract] OR "Autoregressive language model*" [Title/Abstract] OR "Conversational AI" [Title/Abstract] OR  "conversational system*" [Title/Abstract] OR "open-domain dialog*" [Title/Abstract] OR "language model*" [Title/Abstract] OR   "neural language model*" [Title/Abstract] OR  “BERT” [Title/Abstract] OR  “Bard” [Title/Abstract] OR  “Claude”[Title/Abstract] | “Natural language processing”[Mesh] OR “artificial intelligence” [Mesh] | “chatgpt*” [Title/Abstract] OR  "chat generative" [Title/Abstract] OR  "generative AI" [Title/Abstract] OR  “artificial intelligence” [Title/Abstract] OR  “AI” [Title/Abstract] OR  "generative artificial intelligence" [Title/Abstract] OR  “GPT” [Title/Abstract] OR  "Generative Pretrained Transformer" [Title/Abstract] OR  “chatbot*” [Title/Abstract] OR   "Large language model*" [Title/Abstract] OR  "AI language model*" [Title/Abstract] OR  "artificial intelligence language model*" [Title/Abstract] OR  "conversational agent*" [Title/Abstract] OR  "Natural language generation" [Title/Abstract] OR  “NLG” OR "Text generation" [Title/Abstract] OR  "text synthesis" [Title/Abstract] OR  "Transformer language model*" [Title/Abstract] OR "Autoregressive language model*" [Title/Abstract] OR "Conversational AI" [Title/Abstract] OR  "conversational system*" [Title/Abstract] OR "open-domain dialog*" [Title/Abstract] OR "language model*" [Title/Abstract] OR   "neural language model*" [Title/Abstract] OR  “BERT” [Title/Abstract] OR  “Bard” [Title/Abstract] OR  “Claude”[Title/Abstract] OR  “Natural language processing”[Mesh] OR “artificial intelligence” [Mesh] |
| Study type | NOT ("Editorial"[pt] OR  "Comment"[pt] OR  “Review”[pt] OR  “Meta-analysis”[pt] OR  “Systematic review”[pt] OR  “Case reports”[pt] OR  “Observational study”[pt] OR  “Letter”[pt] OR  “News”[pt] OR  “Conference proceedings”  ) |  | NOT ("Editorial"[pt] OR  "Comment"[pt] OR  “Review”[pt] OR  “Meta-analysis”[pt] OR  “Systematic review”[pt] OR  “Case reports”[pt] OR  “Observational study”[pt] OR  “Letter”[pt] OR  “News”[pt] OR  “Conference proceedings” ) |
| Other  (Not Used) | “Turing test” [Title/Abstract] OR  “Efficacy” [Title/Abstract] OR  “Effectiveness” [Title/Abstract] OR  “Simulation” [Title/Abstract] |  | “Turing test” [Title/Abstract] OR  “Efficacy” [Title/Abstract] OR  “Effectiveness” [Title/Abstract] OR  “Simulation” [Title/Abstract] |
