# Supplementary material for: Evaluating Generative AI in Mental Health: Systematic Review of Capabilities and Limitations
Source: JMIR Ment Health. 2025 May 15;12:e70014. doi: 10.2196/70014 (PMC12097452; doi:10.2196/70014)
Supplement: Multimedia Appendix 2 [file mental-v12-e70014-s002.docx]

**Multimedia Appendix 2: Study Characteristics Extraction Table**

| **Author** | **Year** | **Country** | **Users** | **Clinical contet** | **GenAI model** | **Prompting** | **Study design coding** | **Comparison group** | **Therapy-related skills evaluated** |
| --- | --- | --- | --- | --- | --- | --- | --- | --- | --- |
| Razdan et al. | 2023 | US | NA | Erection Dysfunction | ChatGPT-3.5 | Zero shot | Prompt for respnose + human evaluatorm + standardized rating manual | NA | Accuracy, readability, and reproducibility of ChatGPT’s responses |
| Alanezi | 2024 | Saudi Arabia | Participants were outpatients from a hospital in Saudi Arabia. | Anxiety, depression, and behavioral disorders. | ChatGPT-3.5 | No prompt | User+bot interaction | NA | Psychoeducation, emotional support, goal setting and motivation, self assessment and monitoring, resource info, CBT techniques, crisis intervention, psychoteraputic exercises |
| Maurya et al. | 2024 | US | NA | NA | ChatGPT-3.5 | Zero shot | Prompt for respnose + human evaluatorm + standardized rating manual | NA | Psychoeducation, empathy, engagement, clarity, accuracy, relevance, and ethical considerations in response to mental health-related queries. |
| Elyoseph et al. | 2023 | Israel, UK | NA | NA | ChatGPT-3.5 | Zero shot | Prompt for respnose + human evaluatorm + standardized rating manual | Human norms | Emotional awareness (EA) measured via the LEAS, including empathy and the ability to reflect emotions based on scenarios. |
| Elyoseph et al. | 2023 | Israel, UK | NA | Schizophrenia | ChatGPT-3.5, ChatGPT-4, Google Bard, and Claude | Zero shot | Prompt for respnose + human evaluatorm + standardized rating manual | AI, experts, public | Perspectives on schizophrenia recovery, treatment effectiveness, and recovery potential. |
| Gore & Dove | 2024 | UK | Education: counseling and psychotherapy students (MA program) | NA | no specific examples; Questions were on AI in general | No prompt | Survey users regarding their experience and perception of genAI tools | NA | Ethical considerations on AI use in counseling and psychotherapy education |
| B.T & Chen | 2024 | Singapore | NA | Alzheimer's Dementia | ChatGPT-3.5, ChatGPT-4, Google Bard | Chain-of-Thought (CoT) | Prompt for response (transcribed text) + correct diagnosis | Multiple AI models | Recognizing AD (Alzheimer's dementia) |
| Hadar-Shoval et al. | 2023 | Israel | NA | Borderline Personality Disorder, Schizoid Personality Disorder | ChatGPT-3 | Zero shot | Prompt for respnose + human evaluatorm + standardized rating manual | NA | Emotional awareness (EA), mentalizing |
